# Effect of ACTH and hCG on the Expression of Gonadotropin-Inducible Ovarian Transcription Factor 1 (*Giot1*) Gene in the Rat Adrenal Gland

**DOI:** 10.3390/ijms19082285

**Published:** 2018-08-03

**Authors:** Karol Jopek, Marianna Tyczewska, Manjunath Ramanjaneya, Marta Szyszka, Piotr Celichowski, Paulina Milecka, Ludwik K. Malendowicz, Marcin Rucinski

**Affiliations:** 1 Department of Histology and Embryology, Poznan University of Medical Sciences, Swiecickiego 6 Street, 60-781 Poznan, Poland; kjopek@ump.edu.pl (K.J.); maritycz@ump.edu.pl (M.T.); mszyszka@ump.edu.pl (M.S.); pcelichowski@ump.edu.pl (P.C.); paulina.a.grabowska@gmail.com (P.M.); lkm@ump.edu.pl (L.K.M.); 2 Qatar Metabolic Institute, Academic Health System and Department of Medicine, Hamad Medical Corporation, 3050 Doha, Qatar; MRamanjaneya@hamad.qa

**Keywords:** rat, adrenal gland, gene expression, corticotropin, gonadotropin, gonadotropin-inducible ovarian transcription factor-1

## Abstract

Gonadotropin-inducible ovarian transcription factor-1 (*Giot1*) belongs to a family of fast-responsive genes, and gonadotropins rapidly induce its expression in steroidogenic cells of ovaries and testes of rats. Gonadal *Giot1* gene expression is regulated by cyclic adenosine monophosphate (cAMP) -dependent protein kinase A pathway, with essential role of orphan nuclear receptor NR4A1 transcription factor (nuclear receptor subfamily 4, group A, member 1). A recent study reports that *Giot1* is also expressed in adrenals, however, the mechanism of its regulation in adrenal gland is yet to be identified. Therefore, the aim of this study was to characterise the changes in *Giot1* gene expression in male and female rat adrenals using wide range of in vivo and in vitro experimental models. Special emphasis was directed at the *Giot1* gene regulation by ACTH and gonadotropin. In our study, we found that ACTH rapidly stimulates *Giot1* expression both in vivo and in vitro. However, gonadotropin does not affect the adrenal *Giot1* gene expression, presumably due to the low expression of gonadotropin receptor in adrenals. Both testosterone and estradiol administered in vivo had inhibitory effect on *Giot1* gene expression in the adrenals of post-gonadectomized adult rats. Further, our studies revealed that the intracellular mechanism of *Giot1* gene regulation in rat adrenals is similar to that of gonads. As in the case of gonads, the expression of *Giot1* in adrenal gland is regulated by cAMP-dependent signaling pathway with essential role of the NR4A1 transcription factor. The results of our studies suggest that *Giot1* may be involved in the regulation of rat adrenocortical steroidogenesis.

## 1. Introduction

Steroidogenic cells from ovaries, testes, and adrenal cortex are formed during embryonic development from the same progenitor cells derived from adrenogonadal primordium [1,2]. These common ontogenetic origins are reflected in the similarity of adrenal cortex and gonads functions, including the hypothalamic–pituitary feedback regulation and production of steroid hormones [3]. Moreover, essential genes for steroidogenesis (e.g., Star, Cyp11a1, 3BHSD), as well as huge set of transcription factors (e.g., SF-1, DAX-1) are expressed both in adrenals and gonads. A preceding study indicates that these transcription factors play essential role in the regulation of fundamental cellular processes, such as cell fate determination, differentiation, migration, and apoptosis [4,5,6].

Gonadotropin-inducible ovarian transcription factor-1 (*Giot1*) belongs to a family of fast-responsive genes, and its expression is strongly and rapidly induced in vivo by pregnant mare’s serum gonadotropin (PMSG) and human chorionic gonadotropin (hCG) in immature rat ovaries. The gene is also stimulated in vitro by follicle-stimulating hormone (FSH) in rat ovarian granulosa cells [7]. Moreover, the mRNA levels of *Giot1* were temporarily induced in the testicular Leydig cells following PMSG/hCG treatment [8]. The distribution of *Giot1* gene in rats is predominantly restricted to the pituitary, adrenal gland, testis, ovary and hypothalamus [8,9]. It is worth to note that gonadal distribution of *Giot1* overlaps with Ad4- binding protein/steroidogenic factor-1 (Ad4BP/SF-1) and Dax-1 genes, which are essential for sex differentiation and steroidogenesis [10,11].

*Giot1* gene is controlled by cAMP-dependent protein kinase A pathway (cAMP/PKA). Mutational studies of *Giot1* proximal promoter in ovarian granulosa cells identified importance of cAMP response element (CRE) in *Giot1* regulation [7]. Furthermore, the *Giot1* promoter directly interacts with orphan nuclear receptor NR4A1 transcription factor (nuclear receptor subfamily 4, group A, member 1, also known as NUR77), which is controlled by cAMP-dependent pathway [9,12]. 

In ovarian granulosa cells and Leydig’s cells, PMSG and/or hCG stimulates *Giot1* expression. In this context, it could be speculated that the *Giot1* gene is likely to be present in the rat adrenals [8,13]. Using the RNA-seq technology, Yu [13] studied gene expression pattern in 11 organs of rats of the Fischer strain in four developmental stages: juvenile (2 weeks), adolescence (6 weeks), adult (21 weeks) and aged (104 weeks). Among the organs studied (adrenal, brain, heart, kidney, liver, lung, muscle, spleen, testes, thymus, uterus), expression of the *Giot1* gene in adrenals was several times higher than the other organs studied. Also, we have previously shown that the *Giot1* gene is highly expressed in fasciculata/reticularis zones of female adult adrenals compared to males [14]. However, the mechanism of regulation of adrenal *Giot1* gene expression is not known. Furthermore, there are inconsistent data on the expression of luteinizing hormone/choriogonadotropin receptor (*Lhcgr*) in rat adrenocortical cells. Conversely, ACTH, the main adrenal cortex regulator, also stimulates the cAMP/PKA signaling pathway through melanocortin 2 receptor (MC2R) [15]. These findings suggest that the expression of the *Giot1* gene in the adrenal gland can be regulated by ACTH, at least partly through the intracellular signaling pathway through which gonadotropins also act.

In the context of the above information, the aim of the study was to characterize the expression of *Giot1* gene in adrenals of adult male and female rats. Special emphasis was directed to the regulation of gene expression by ACTH and gonadotropin, as well as by sex hormones. The findings from our study suggest that the main factors regulating the expression of *Giot1* gene in rat adrenals are ACTH and sex hormones.

## 2. Results

### 2.1. Analysis of RNA-Seq Dataset Obtained from Adrenals of Male and Female Rats during Postnatal Ontogenesis

Experimental data were extracted from Gene Expression Omnibus database, accession number: GSE53960 (available at https://www.ncbi.nlm.nih.gov/geo/query/acc.cgi?acc=GSE53960) (accessed date: 03 August 2018) which was deposited by Yu [13].

**Figure 1:**
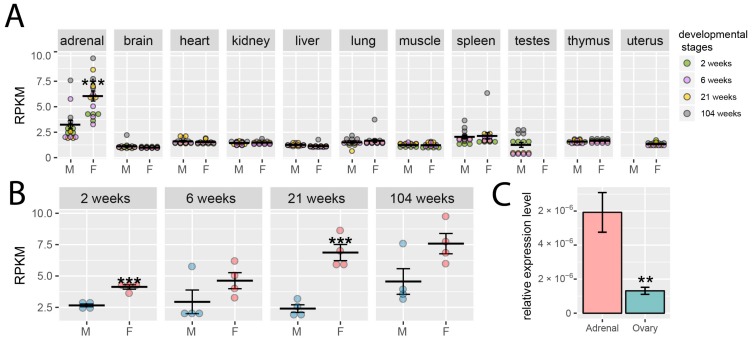
(**A**) Expression levels (RPKM—reads per kilobase million) of *Giot1* gene in 11 organs of male and female rats of the Fischer strain in four developmental stages: juvenile (2 weeks), adolescence (6 weeks), adult (21 weeks) and aged (104 weeks). Color of the circle corresponds to the appropriate development stage; (**B**) expression levels of *Giot1* gene in entire adrenal glands of Fischer 344 male and female rats in course of ontogenesis. The analysis of data published by Yu [13]. Experimental data was obtained from Gene Expression Omnibus database, accession number: GSE53960 (available at https://www.ncbi.nlm.nih.gov/geo/query/acc.cgi?acc=GSE53960) (accessed date: 3 August 2018); (**C**) comparison of the level of expression of the *Giot1* gene in adrenals and ovaries of adult rats of the Wistar strain. Expression measured by the quantitative polymerase chain reaction (qPCR) method (*n* = 4). Data expressed as means ± SEM. Each circle represents a single rat. Statistically significant differences in relation to control group (Student’s *t*-test): *** *p* < 0.01, ** *p* < 0.02

Using the RNA-seq method, these authors demonstrated that *Giot1* expression was highest in rat adrenals compared to other tissues studied and female rats had higher *Giot1* expression in adrenals compared to males, whilst sexual dimorphism did not alter *Giot1* expression in other organs (brain, heart, kidney, liver, lung, muscle, spleen, testes, thymus, uterus) (Figure 1A). A detailed analysis of the gene expression pattern in adrenals revealed higher levels of its expression in females than in males, which is statistically significant in juvenile and adult rats (Figure 1B). Since the ovaries have not been included in the Yu et al. experiment, we decided to compare the basal level of *Giot1* gene expression in ovaries in relation to female adrenals. In sexually mature rats, the adrenal expression of the *Giot1* gene is significantly higher than in ovaries (Figure 1C). Taking into account both Yu et al. and our results it should be noted that the adrenal gland is characterized by the highest expression of *Giot1* gene from all of analyzed rat organs.

### 2.2. Effects of ACTH on Giot1 mRNA Level in Rat Adrenal Glands

**Figure 2:**
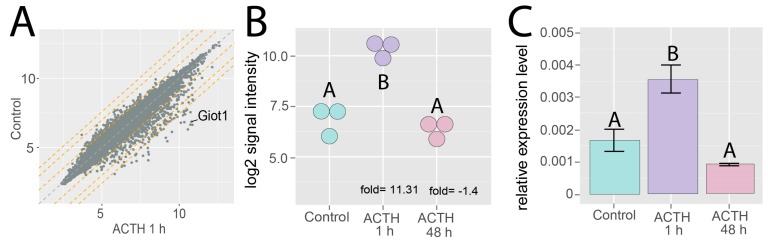
Effect of ACTH (adrenocorticotropic hormone) on *Giot1* gene expression in adult male adrenals. Rats were intraperitoneally injected with acute (ACTH 1 h, 5 µg /rat injected 1h before decapitation) or prolonged quadruple injections of ACTH with 12 h intervals (ACTH 48 h). Control group of rats were injected with physiological saline solution and studied at 60 min post-injection. (**A**) Scatter plot from microarray transcriptome studies of adrenals from control vs 1 h ACTH treatment experimental group. The average expression value of the *Giot1* gene is shown. Orange dotted lines denote expression above 2, 4, 6 fold changes; (**B**) data extracted from normalized gene expression dataset of Affymetrix Rat Gene 2.1 ST Array, presented as a dot plot in log2 signal intensity scale. Expression fold change was calculated in relation to control group; (**C**) qPCR validation of microarray results. Bars represent means ± SEM (*n* = 3). Statistical analysis of the data was performed by using one-way analysis of variance (ANOVA) followed by Tukey’s honest significant difference test (Tukey’s HSD test). Groups sharing the same letter are not significantly different according to Tukey’s HSD test.

Using microarray expression analysis, we found that within 1 hour following i.p. ACTH administration, the *Giot1* gene expression increased significantly in the rat adrenal gland and is one of the most up-regulated gene (Figure 2A) The expression of *Giot1* increased by 13.31 times compared to the control group (Figure 2B). Prolonged quadruple injections of ACTH in 12 h intervals (ACTH 48 h) do not significantly change the expression of *Giot1* in relation to control group. These findings was further confirmed by qPCR method (Figure 2C).

**Figure 3:**
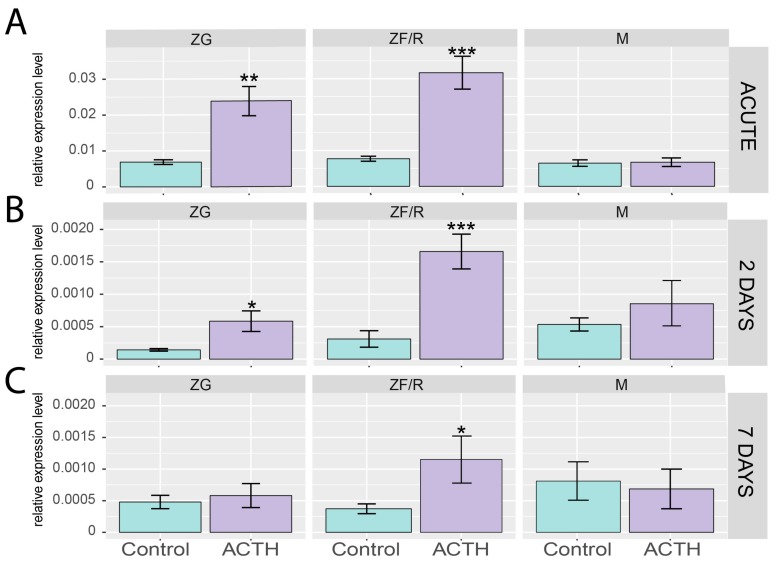
ACTH effects on *Giot1* gene expression in zona glomerulosa (ZG), zona fasciculata/reticularis (ZF/R) and adrenal medulla (M) of adult male rats. Expression of *Giot1* was measured by qPCR method after 1 h of i.p. ACTH administration (**A**) or long-term infusion of corticotropin (ALZET microosmotic pumps) for 2 days (**B**) and 7 days (**C**) (ACTH infusion rate 2 nmol/24 h/100 g). Bars represent means ± SEM (*n* = 4). Statistically significant differences in relation to control group of each adrenal zones (Student’s *t*-test): * *p* < 0.05; ** *p* < 0.02; *** *p* < 0.01.

In the next series of experiments, we compared the effect of acute administration of ACTH (1 h, Figure 3A) along with chronic long-term infusion of corticotropin (ALZET micro osmotic pumps) for 2 (Figure 3B) and 7 days (Figure 3C) (ACTH infusion rate 2 nmol/24 h/100 g). All the compartments of adrenal glands (ZG, ZF/R and M) were isolated to profile for *Giot1* expression (Figure 3A–C). The expression of the *Giot1* gene was observed in all adrenal compartments. In both acute and prolonged treatment (2 and 7 days) groups, the basal expression (control) of *Giot1* gene was relatively similar in all of the adrenal compartments per each of the study groups.

The stimulatory effect of ACTH on the *Giot1* gene expression was the most significant following acute stimulation, and the effects gradually decreased by 2- and 7 days of chronic studies. Within the zona glomerulosa (ZG), ACTH led to an increase in *Giot1* expression after acute and prolonged 2-day administration, whereas it did not alter the expression in ZG after 7-day hormone infusion. The most potent stimulating effect of ACTH on the expression of *Giot1* gene was observed in ZF/R at all time points tested. The lowest increase was observed following 7-day infusion of ACTH. In the entire study, ACTH did not change the expression of *Giot1* gene in M.

### 2.3. Effects of Gonadectomy and Testosterone or Estradiol Replacement on the Expression of the Giot1 Gene in Rat Adrenal Gland

**Figure 4:**
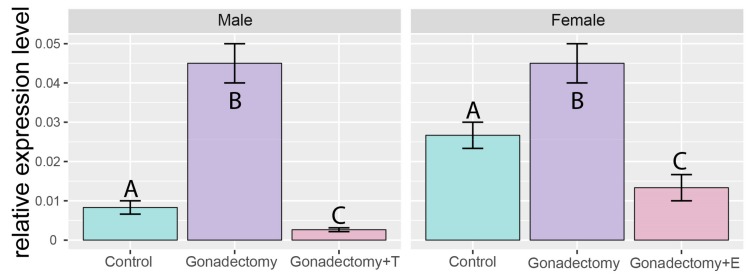
Effects of gonadectomy and testosterone (T) or estradiol (E) replacement on *Giot1* gene expression in rat adrenals measured by qPCR method. Bars present means ± SEM (*n* = 5). Statistical analysis of the data was performed by using one-way analysis of variance (ANOVA) followed by Tukey’s HSD test. Groups sharing the same letter are not significantly different according to Tukey’s HSD test.

It is well-known that gonadectomy increases secretion of gonadotropins (FSH and LH). Therefore, in further experiment, we investigated the effect of gonadectomy and gonadal hormone replacement on the *Giot1* gene expression in adrenal glands of adult rats. The expression of *Giot1* gene in adrenals increased significantly after both orchiectomy (ORX) and ovariectomy (OVX). In ORX rats supplementation with testosterone and in OVX rats with estradiol decreased the level of expression of the gene, compared to the sham surgery rats (Figure 4).

### 2.4. Effects of ACTH and hCG on the Expression of Giot1 Gene in Rat Primary Adrenocortical Cells

**Figure 5:**
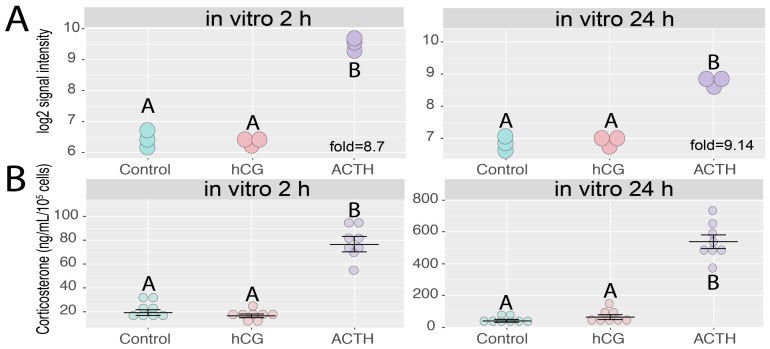
Effect of ACTH and hCG on *Giot1* gene expression in primary adrenocortical cells. (**A**) Cells were collected for RNA isolation 2 and 24 hours after administration of the tested compounds. Data extracted from normalized gene expression dataset of Affymetrix Rat Gene 2.1 ST Array, presented as a dot plot in log2 signal intensity scale. Expression fold change was calculated in relation to control group (*n* = 3); (**B**) effect of hCG and ACTH on corticosterone secretion in primary rat adrenocortical cells. At day 4 of culture, cells were exposed to hCG (10^−7^ M), or ACTH (10^−7^ M). Cell culture media were collected at 2 h and 24 h following drug treatment. Corticosterone concentration was determined by ELISA. Data are presented as means ± SEM, *n* = 8. Statistical analysis of the data was performed by using one-way analysis of variance (ANOVA) followed by Tukey’s HSD test. Groups sharing the same letter are not significantly different according to Tukey’s HSD test.

The direct effect of ACTH and gonadotropin on adrenal *Giot1* gene expression was investigated using primary culture of rat adrenocortical cells. For this purpose, the adrenocortical cell cultures, after reaching 80% of confluence, were incubated for 2 and 24 h with hCG or ACTH. *Giot1* expression was analyzed using microarrays. The results demonstrate that ACTH strongly stimulated expression of *Giot1* after both 2 and 24 h (fold = 8.7 and 9.14 respectively). In contrast to potent stimulatory effect of ACTH, *Giot1* gene expression was not altered by hCG treatment (Figure 5A). Further, we examined effects of two and 24-h hCG and ACTH treatment on corticosterone secretion in primary rat adrenocortical cells. As expected, ACTH significantly stimulated corticosterone secretion whereas hCG did not affect corticosterone secretion (Figure 5B).

**Figure 6:**
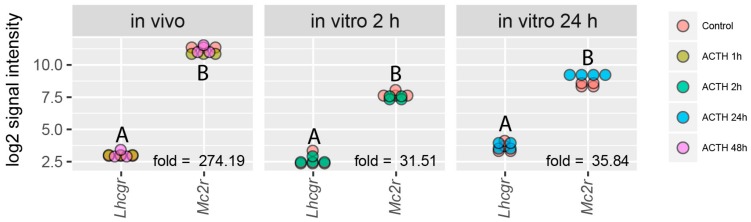
Expression of luteinizing hormone/choriogonadotropin receptor (*Lhcgr*) and melanocortin 2 receptor (*Mc2r*) in rat adrenals (in vivo—after i.p. ACTH injections) or in primary rat adrenocortical cells (in vitro 2 h, in vitro 24 h). Data extracted from normalized gene expression data set of Affymetrix Rat Gene 2.1 ST Array, presented as a dot plot in log2 signal intensity scale. Expression fold change was calculated in relation to *Lhcgr* gene. Statistical analysis of the data was performed by using one-way analysis of variance (ANOVA) followed by Tukey’s HSD test. Groups sharing the same letter are not significantly different according to Tukey’s HSD test.

We hypothesised that the lack of hCG effect on the corticosterone secretion and *Giot1* gene expression might be due to relatively low expression of the *Lhcgr* in rat adrenal gland. To test this hypothesis, the log2 normalized signal intensities for *Lhcgr* and *Mc2r* were extracted from all of performed microarray experiments. Then, normalized signal intensities for *Lhcgr* and *Mc2r* were compared with each other with estimation of fold changes (in relation to *Lhcgr* gene). Normalized signal intensities and fold change values are shown in Figure 6. In both in vivo (after i.p. ACTH injections) and 2 and 24 h in vitro experiments the expression of *Lhcgr* gene was relatively low, and its value measured in log2 normalized signal intensities scale reached about 3. Expression of *Mc2r* was the highest in the in vivo studies, reaching a log2 normalized signal intensity value of approximately 11. The lower *Mc2r* expression values were detected in the in vitro experiments in the range of 7.5–8. Nonetheless, expression of *Mc2r* in any of the analyzed comparisons was considerably higher than that of *Lhcgr*. Regarding the fold change values, the *Mc2r* expression was 274 times higher in the in vivo experiments, as well as 31.5 and 36 times higher in the in vitro experiments following 2 and 24 h stimulation respectively, in relation to *Lhcgr* (Figure 6).

**Figure 7:**
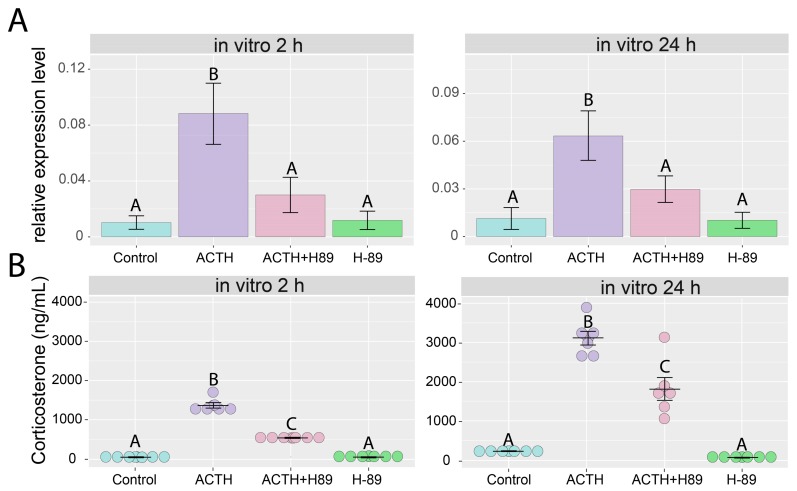
Effect of protein kinase A inhibitor—H-89 (10^−5^ M) on basal and ACTH (10^−7^ M) stimulated *Giot1* gene expression in primary culture of rat adrenocortical cells. (**A**) The expression of *Giot1* was determined by qPCR method. Cells were collected for RNA isolation 2 and 24 h following administration of the tested compounds. Bars represent means ± SEM (*n* = 5); (**B**) effect of H-89 (10^−5^ M) on basal and ACTH (10^−7^ M) stimulated corticosterone output in primary cultures of rat adrenocortical cells. Cell culture media were collected at 2 and 24 h after administration of the tested compounds. Corticosterone concentration was determined by ELISA. Data are presented as means ± SEM (*n* = 6). Statistical analysis of the data was performed by using one-way analysis of variance (ANOVA) followed by Tukey’s HSD test. Groups sharing the same letter are not significantly different according to Tukey’s HSD test.

Our results show that ACTH increases the expression of *Giot1* gene in rat adrenals. However, the mechanism of this action is not clear. It is known that in adrenals ACTH stimulates cAMP-dependent protein kinase A through MC2R receptor, which in turn stimulates target genes expression. To clarify whether the activated ACTH signaling pathway cAMP-dependent protein kinase A is involved in the regulation of the *Giot1* gene in the adrenal gland, we performed further studies using primary adrenocortical cells. In this experimental model preincubation with H-89, a specific PKA inhibitor significantly inhibited the ACTH induced expression of the *Giot1* gene and corticotropin-induced corticosterone secretion (Figure 7A,B). This indicates that ACTH dependent control of *Giot1* expression occurs by cAMP/PKA dependent signal pathway. The H89 alone did not affect the expression of the *Giot1* gene (Figure 7).

**Figure 8:**
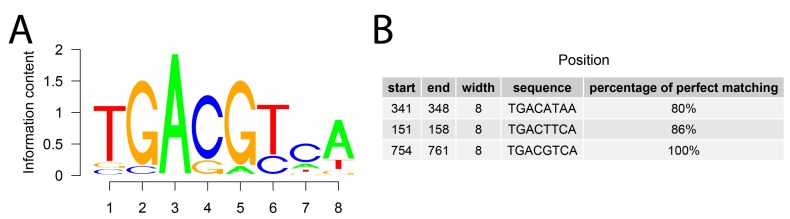
Analysis of the *Giot1* gene promoter sequence for the presence of conservative CREB1 (cAMP responsive element binding protein 1) binding sequence. (**A**) Sequence logo based on CREB1 frequency matrix obtained from Jaspar—a database of transcription factor binding profiles (id: MA0018.2, http://jaspar.genereg.net/matrix/MA0018.2/ as at 03 August 2018); (**B**) table of potential binding sites of CREB1. The binding position within 1000 nucleotides of the promoter was indicated by start and end values. Width, promoter of sequence matched to frequency matrix and percentage of perfect matching were also shown.

The regulation of *Giot1* gene expression by cAMP has been confirmed using a bioinformatics approach based on the analysis of the *Giot1* gene promoter sequence for the presence of conservative CREB1 (cAMP responsive element binding protein 1) binding sequence. The sequence logo based on CREB1 frequency matrix is shown at Figure 8A. Perfect percentage matching limit was set at 80% similarity to the CREB1 frequency matrix. We have shown that within 1000 bp of the *Giot1* gene promoter, there are three potential CREB1 binding sequences. One of them in the position 754–761 bp. of selected 1000 bp. sequence, being a perfect 100% match to the CREB1 frequency matrix pattern (Figure 8B).

**Figure 9:**
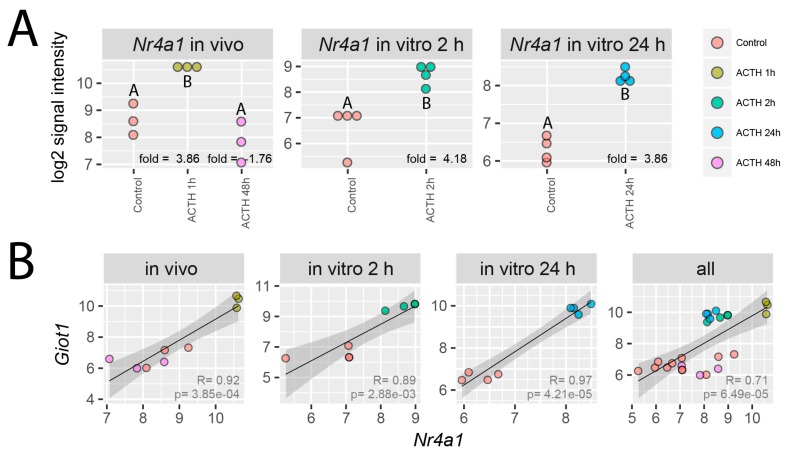
Expression of *Nr4a1* in rat adrenals (in vivo) or in primary culture of rat adrenocortical cells (in vitro 2 h, in vitro 24 h). (**A**) Data extracted from normalized gene expression data set of Affymetrix Rat Gene 2.1 ST Array, presented as a dot plot in log2 signal intensity scale. Expression fold change was calculated in relation to control group. Statistical analysis of the data was performed by using one-way analysis of variance (ANOVA) followed by Tukey’s HSD test. Groups sharing the same letter are not significantly different according to Tukey’s HSD test; (**B**) linear correlation of *Giot1* vs *Nr4a1* gene expression in rat adrenals (in vivo) or in primary culture of rat adrenocortical cells (in vitro 2 h, in vitro 24 h) or in all of analyzed samples. Pearson correlation coefficient *R*-values and p-values are shown. 95% confidence interval is marked.

Preceding studies demonstrate that the expression of *Giot1* is also regulated by orphan nuclear receptor NUR77 encoded by the *Nr4a1* gene [12]. Therefore, microarray data for *Nr4a1* gene were extracted from all of analyzed experimental groups. Fold changes withlog2 normalized signal intensity values were presented in Figure 9. In all of experimental conditions expression profile of *Nr4a1* gene corresponds to the expression of *Giot1*. Analogous to the *Giot1* gene, the expression of *Nr4a1* was also significantly increased in 1 hour following i.p. ACTH administration and after exposure of cells 2 and 24 h to ACTH in our in vitro model (Figure 9A). Expression of *Nr4a1* in each experimental group strongly correlated with expression of *Giot1* gene. Correlation analysis using the data from all experimental groups showed very strong positive correlations with the coefficient R = 0.71 and *p* = 6.49 × 10^−5^ (Figure 9B).

### 2.5. Bioinformatic Ananalyses Concerning the Other Species

**Figure 10:**
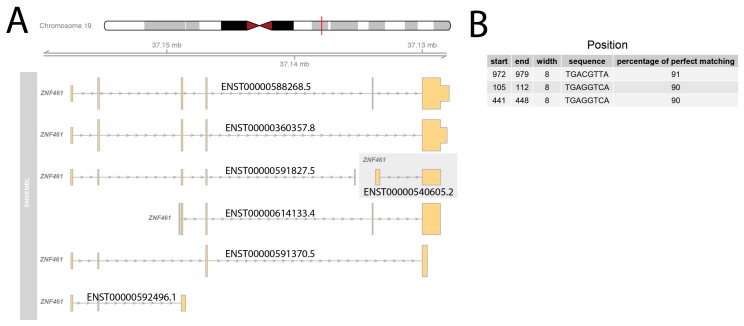
Analysis of the human *ZNF461* (zinc finger protein 461) gene promoter sequence for the presence of conservative CREB1 (cAMP responsive element binding protein 1) binding sequence. (**A**) Structure of human *ZNF461* splice variants. The exons are highlighted in orange. The exons or their noncoding part are lower in shape compared to the high in shape of the coding exons. ENSEMBL accession numbers are shown. The grey box indicates a transcriptional splice variant with potential binding sites of CREB1; (**B**) table of potential binding sites of CREB1 on ENST00000540605.2 promoter. The binding position within 1000 nucleotides of the promoter was indicated by start and end values. Width, promoter of sequence matched to frequency matrix and percentage of perfect matching were also shown.

As *ZNF461* is considered to be the human homologue of the rat *Giot1* gene [16], it seems that *ZNF461* should also have CREB1 binding motif in the sequence of promoter. For this reason, 1000 nt. promoter sequence from the seven transcriptional variants of *ZNF461* gene were analyzed in a similar manner as described above. Analysis of seven promoters from *ZNF461* transcriptional splice variants revealed that only one of the *ZNF461* transcript isoform contains strong three positions with potential CREB1 binding site (Figure 10). This variant is composed of two exons forming a nonprotein coding transcript. None of the other transcriptional variants had the potential of CREB1 binding sequences. This result suggests that *ZNF461* expression is not regulated by cAMP.

**Figure 11:**
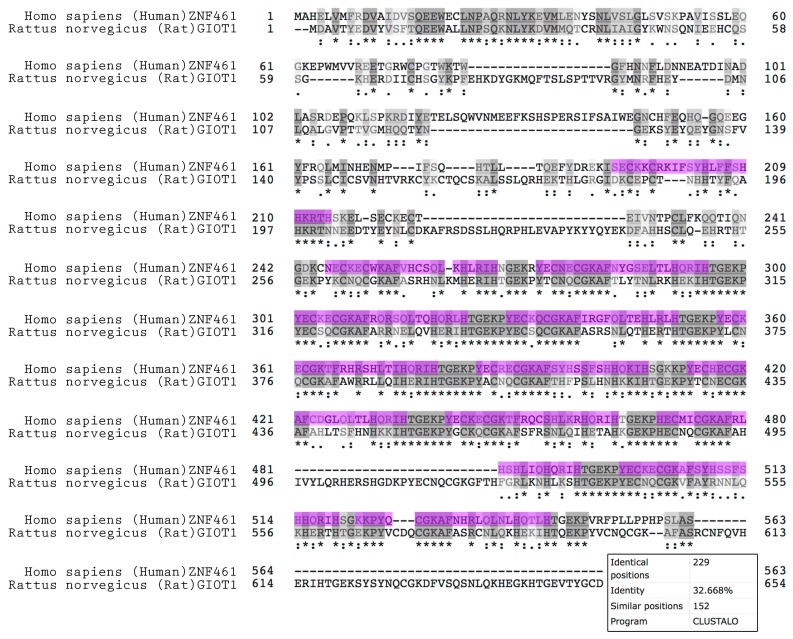
Multiple sequence alignment of the following protein sequences: *Homo sapiens* (human) ZNF461, *Rattus norvegicus* (rat) GIOT1. Identical sequences are marked with a dark grey color and an asterisk. Similar sequences are indicated by a light grey color and a colon or dot depending on the degree of similarity. Conservative zinc fingers domains of human ZNF461 were marked in purple.

Only rat GIOT1 sequence is available in protein databases. However ZNF461 protein sequences are available for many other species. These sequences, together with GIOT2 (rat GIOT1 like protein) and human ZNF460 were retrieved from UniProt protein database (https://www.uniprot.org) to compare their amino acid sequence homologues. Interestingly, there is no known sequence of both GIOT1 or ZNF461 proteins for mice. In total, ten sequences from different species were analyzed for multiple sequence alignment. The highest identity and similarity of the analyzed sequences concerned conservative zinc finger domains (Figure S1A). Phylogenetic tree generated from a multiple sequence alignment demonstrated the presence of three separate clusters of the compared sequences (Figure S1B). The first cluster consisted of the most diverse protein sequence—ZNF460, the second one consisted of GIOT1 and GIOT2 rat proteins, while the third one contained all proteins described as ZNF461. The obtained results indicate significant discrepancies in the sequence of rat GIOT and ZNF461 of other species. A direct comparison of the rat GIOT1 protein sequence and human ZNF461 confirmed the low homology for the analyzed proteins (Figure 11). Out of 654 amino acids of GIOT1 protein, only 229 aa had identical positions and 152 aa similar positions among the sequences compared. The calculated percentage of identity, according to the Clustal Omega program, was only 32.668% which indicates low homology of the compared proteins.

## 3. Discussion

The *Giot1* gene belongs to the novel members of the zinc finger transcription factors family. It contains two well-described domains: consists of 14 (Cys)_2_-(His)_2_-type zinc finger motifs and a Krüppel associated box-A (KRAB-A) domain [8,16]. The (Cys)_2_-(His)_2_-type zinc finger motif is responsible for DNA-binding properties of transcription factors, whereas the KRAB-A domain has been shown to possess transcriptional repressor activity [17,18]. *Giot1* expression levels are regulated rapidly in a presence of adequate stimulus, which allows qualifying *Giot1* to group of fast, responsive factors. In immature rat ovaries, the expression of *Giot1* increases rapidly and strongly after administration of pregnant mare’s serum gonadotropin (PMSG) or human chorionic gonadotropin (hCG). Expression of this gene also increases strongly after the addition of a follicle-stimulating hormone (FSH) to culture of ovarian granulosa cells. Moreover, it has been shown that in vitro levels of mRNA *Giot1* in Leydig cells increase significantly after addition to the medium of pMSG/hCG [7,8,12]. On the other hand, Mizutani et al., using the RT-PCR method, showed that *Giot1* is expressed in the rat adrenal glands [8]. Taking into account the common origin of gonadal steroidogenic and adrenocortical cells as well as similar intracellular mechanisms that regulate steroidogenesis, as well as the fact that *Giot1* expression was the highest in the adrenal glands among all analyzed organs, we hypothesised that expression of *Giot1* gene in adrenal cortex can also be regulated by ACTH. Indeed, in our in vivo studies, after acute 1-hour administration of ACTH, significantly increasing of *Giot1* gene expression in the adrenal glands of the rat was determined both by microarrays and qPCR techniques. In the model of prolonged i.p. ACTH injection, in which *Giot1* gene expression was determined at 12 h since the last administration of ACTH, we did not observe the stimulating effect of ACTH on *Giot1* gene expression. Similar observation was found in the context of pMSG and hCG effect on the *Giot1* gene expression in ovaries of immature rats. In these studies, *Giot1* transcript was temporally induced in the ovaries of immature rats after administration of PMSG or hCG. The preceding study, using northern blot technique, showed that *Giot1* gene expression began to increase within 3 h after administration, reaching maximal level at 6 h and returned to basal levels by 12 h after pMSG or hCG administration [8]. Based on the above results and our data on the effect of ACTH in vivo on the expression of *Giot 1* gene in the adrenal gland, it may be suggested that both in gonads and in the adrenal glands of the rat *Giot1* belongs to the group of genes rapidly and temporarily reacting to the appropriate ligand. However, in the prolonged ACTH infusion model, using ALZET micro osmotic pump for two and seven days, expression of *Giot1* was increasing in adrenal cortex, but the most potently in ZF/R. Expression of *Giot1* gene in adrenal medulla was also detected, but ACTH does not change it. It is clear that the ZF mainly produces glucocorticoids in response to ACTH interaction with melanocortin-2-receptor (MC2R). This interaction results in stimulation of adenylyl cyclase, which catalyses the conversion of ATP to cAMP [19,20]. Therefore, the strongest effect observed in ZF is not surprising and suggests that the stimulus for changes in expression of the *Giot1* gene originates from the interaction of ACTH with the MC2R receptor.

Our second hypothesis assumed that gonadotropins, analogous to gonads regulate the adrenal expression of the *Giot1* gene. This hypothesis was verified using two experimental models. We studied the effect of elevated gonadectomy-induced concentrations of pituitary gonadotropins on the expression of the *Giot1* gene. In both males and females, gonadectomy increases the expression of the *Giot1* gene in the adrenal glands. This effect was reversed with testosterone or estradiol replacement, respectively, leading to a significant decrease in *Giot1* gene expression. Currently, gonadectomy is considered to be a classical model of in vivo gonadotropin elevation with inhibin decreasing. However, a good deal of evidence suggests that gonadectomy with sex hormones replacement exerts multidirectional effect also on the function of HPA axis, affecting CRH and/or ACTH synthesis and secretion [21,22,23,24]. In our previous studies, we have shown that gonadectomy, with subsequent sex hormones supplementation in adult rats of both sexes, affects the expression of several adrenal genes, belonging among others to cholesterol homeostasis, lipid metabolic process and response to cAMP gene ontological groups [25].

In subsequent experimental model, we demonstrated that 2 and 24 h exposure of the rat’s adrenocortical cells to hCG in primary culture does not affect corticosterone secretion or the expression of the *Giot1* gene. In the available literature, the regulation of physiological adrenal function by gonadotropins is the subject of many controversies [3]. Majority of the studies shows that the adrenal gland might be responsive to LH/hCG stimulation, leading to hyperactivation of the organ in adrenocortical sex hormones secretion. In this aspect, it was shown that hCG administration to newborns leads to an increase of adrenal DHEA secretion [26]. hCG also stimulates dehydroepiandrosterone (DHEA) secretion in isolated human fetal zone of adrenal glands [27]. Conversely, hCG added to a human adult adrenal cell suspension does not affect adrenocortical sex hormones secretion [28]. However, other evidence indicates an indirect role of gonadotropins in control of adrenocortical steroidogenesis. For example, the production of adrenal androgens, which starts to grow during the adrenarche, reaches adult levels during puberty without a concomitant increase in ACTH [29]. At the same time intervals, cortisol level remained unchanged. Currently, limited data are explaining the role of hCG in activation of adrenal steroidogenesis leading to production of glucocorticoids. In this context O’Connell et al., demonstrated that hCG at 10^−7^ and 10^−6^ M concentrations significantly increased cortisol secretion from freshly isolated guinea pig adrenocortical cells [30]. Also, data from transgenic mice with elevated LH secretion indicate that corticosterone synthesis increases in these mice [31]. However, our data do not support these findings. In our study, hCG did not affect neither corticosteroid secretion or the expression of the *Giot1* gene in rat adrenocortical cells. We assumed that the lack of response to hCG stimulation in adrenal cells results from either absence or minimal expression of *Lhcgr* receptor on the surface of rat adrenocortical cells. Indeed, the data obtained from our microarray studies revealed that in both in vitro and in vivo experiments, expression of *Lhcgr* is relatively low. It should be noted that the *Lhcgr* expression is almost negligible compared to *Mc2r* in rat adrenals.

Furthermore, data extracted from our microarray studies indicate that *Mc2r* expression is 274 times higher than *Lhcgr* in adrenals used in in vivo experiments and 31–35 times elevated in in vitro experiments. In accordance with our study, previous studies have demonstrated lower *Lhcgr* expression in human and mouse adrenal cortex [31,32,33]. Low *Lhcgr* expression was also reported in fetal adrenal cortex and the adrenals of patients with pituitary-dependent Cushing’s disease [33,34,35,36]. Many researchers have found the *Lhcgr* receptor protein and/or mRNA in some of some pathological conditions in human adrenal glands, such as adrenocortical adenomas, carcinomas and macronodular adrenal hyperplasia [34,36,37,38,39,40].

Incubation of primary adrenocortical cells with ACTH has shown that our cells were responsive to adrenocortical steroidogenesis stimulation. Additionally, we demonstrated that stimulation of adrenal hormone biosynthesis and secretion is associated with an increase in adrenal *Giot1* gene expression; therefore, we have proved that ACTH directly stimulates expression of the *Giot1* gene in rat adrenal gland. ACTH stimulated *Giot1* gene expression in adrenal gland but not by gonadotropin. However, the intracellular mechanism of this stimulation seems to be similar to that found in the granulosa or Leydig cells. Gonadotropins and ACTH can act on their target cells by increasing a cAMP level [7,41]. Thus, they play critical role in the induction of various genes through activation of cAMP-dependent signaling pathway [8,15,42]. In our study, stimulating effect of ACTH on the expression of the *Giot1* gene was inhibited by administration of specific cAMP inhibitor H-89. It suggests that cAMP-dependent pathway regulates the *Giot1* gene in the rat adrenal gland. Recent studies have shown that *Giot1* gene expression is also upregulated in paraventricular and supraoptic nuclei of rat after osmotic stimulus. This effect was also mediated by cAMP-dependent pathway [9]. In this regard, analysis of the 1000 bp upstream region of *Giot1* promoter revealed there are potentially three cAMP-response element binding sites, with one of them being a 100% match of a site. Our promoter analysis is in line with previous detailed mutational studies of the *Giot1* proximal promoter region, where we identified an important role of cAMP response element in *Giot1* gene expression activation [7]. Furthermore, the *Giot1* promoter region has been identified as a target of orphan nuclear receptor NR4A1 (also known as NUR77 or NGFIB) that has been classified as immediate-early gene induced rapidly but transiently by variety of stimuli [12,43]. Electrophoretic mobility shift and chromatin immunoprecipitation assays revealed that NR4A1 directly binds to the *Giot1* promoter [12]. In human adrenal NR4A1 regulates expression of 3β-hydroxysteroid dehydrogenase type 2 (HSD3B2)-steroid-metabolising enzyme that is essential for adrenal production of mineralocorticoids and glucocorticoids [44].

It has been reported that the NR4A1 induced by high glucose level leads to stimulation of CYP11B2 gene expression and aldosterone production of human adrenal H295R cells [45]. The results of our study showed a strong positive correlation between adrenal expression of *Giot1* and *Nr4a1*, which indirectly confirms the relationship between the studied genes. Furthermore, it has been demonstrated that *Giot1* represses the steroidogenic factor-1 (SF-1; NR5A1) transactivation [12]. SF-1 is the key transcription factor controlling steroidogenesis that regulates number of genes involved in the biosynthesis of steroid hormones [46,47,48]. It is expressed in all zones of the adrenal cortex, ovary, testis, hypothalamus and anterior pituitary gland [49]. Activation of *Giot1* gene expression by NR4A1, leads to repression of SF-1 transactivation and this findings allows to assume that *Giot1* may participate in regulation of rat adrenocortical steroidogenesis. However, further functional studies are required to explain the *Giot1* role in adrenal gland using knock down and/or overexpression studies. A significant limitation of the proposed studies is the lack of confirmation of expression changes in the *Giot1* at the protein level using western blot method. Such an attempt was undertaken during the experimental procedure, however, we used commercially available antibody against GIOT1 (GIOT1 Antibody (C-12): sc-398187, Santa Cruz Biotechnology, Santa Cruz, CA, USA). This antibody was of poor quality, resulting in a number of nonspecific bands. Therefore, the changes in *Giot1* expression at protein level should be also confirmed in the further studies.

Until now, the research on the physiological role of *Giot1* was carried out exclusively on the rat model. Dai et al. described that human ZNF461 shows high similarity with the rat GIOT1, and suggested that ZNF461 is a human GIOT1 homolog [16]. However, this statement seems to be controversial. In our bioinformatic analyses of *ZNF461* promoters, we demonstrated that there is no CREB binding sequence on promoters from protein coding isoforms. In addition, we demonstrated that GIOT1 rat protein has a low amino acid sequence homology with ZNF461. Dai et al. reported that there is 68% identity at the amino acid level between ZNF461 and rat GIOT1 [16]. Our alignment results of GIOT1 and ZNF461 revealed only 32,668% of identity. Additionally, it was shown that human ZNF461 has a different organ distribution. It is widely expressed, with highest levels in liver, kidney, pancreas, thymus, and small intestine. In view of the above, it seems that ZNF461 and GIOT1 are rather divergent genes/protein. Currently there is no sequence of mouse GIOT1 homologue available in the protein databases, however because of their close evolutionary relationship, it seems very likely that such a homologue exists.

## 4. Materials and Methods

### 4.1. Animals and Reagents

Wistar rats were bred in our Laboratory Animal Breeding Centre, Department of Toxicology, Poznan University of Medical Sciences (Poznan, Poland). The animals were kept under standardized light conditions (14:10 h light/dark cycle, illumination onset at 6.00 a.m.) at 23 °C, 50–60% air humidity, 8–10 air changes per hour (mechanical, via HEPA filters) and maintained on a standard diet with free access to tap water. Animals were decapitated between (11–12 a.m.) and studied organs were promptly removed. The number of rats, their sex, age, and body mass used in the current study are given in the descriptions of the individual experiments. The study protocols were approved by the Local Ethics Committee for Animal Studies in Poznan (protocols No. 11/2015 and 75/2016, 06/March/2015). ACTH (Synacthen) was purchased from Novartis (Basel, Switzerland), testosterone (Testoviron-Depot) from Schering AG (Berlin, Germany), estradiol (Estradiol-Depot) from Jenapharm (Jena, Germany) and H-89 dihydrochloride hydrate (Catalog Number B1427) and hCG (Chorionic gonadotropin human Catalog Number CG10, lyophilized powder) from Sigma-Aldrich (St. Louis, MO, USA). Other reagents, unless stated otherwise, were purchased from Sigma-Aldrich (St. Louis, MO, USA) or from Avantor Performance Materials Poland S.A. (Gliwice, Poland).

### 4.2. In Vivo Experiments

#### 4.2.1. Effects of ACTH on *Giot1* mRNA Level in Rat Adrenal Glands

A—acute and prolonged ACTH injections. The experiments were carried out on adult male rats (12 weeks old; body weight: 120–150 g; 3 rats per group). In acute experiment, rats were intraperitoneally (i.p.) administered with ACTH (5 µg/rat) 1h before decapitation. In prolonged experiment rats were administered subcutaneously (s.c.) with ACTH (5 µg/rat) at hours 0, 12, 24 and decapitated 12 h after the last injection. Control group of rats were injected with physiological saline solution (0.2 mL/rat) and studied 60 min post-injection.

B—comparison of the bolus ACTH administration and long corticotropin infusion on the expression of the *Giot1* gene in rat adrenal compartments. These experiments were carried out on adult male rats (12 weeks old; body weight: 120–150 g; 4 rats per group). In acute experiment rats were i.p. administered with ACTH (5 µg/rat) 1 h before decapitation. Other groups of rats were given ACTH in ALZET microosmotic pumps (models 1003 and 2001 for 2 and 7 days infusions, respectively). Mini pumps containing ACTH were implanted s.c., (under ketamine (100 mg/kg, i.p.) and xylazine (10 mg/kg, i.p.) anesthesia), the infusion rate was 2 nmol/24 h/100 g. Pumps filled with 0.9% saline were implanted into the control groups. After decapitation, the adrenal glands were promptly removed and freed of adherent adipose tissue. Subsequently, under a stereomicroscope, glands were decapsulated to separate the zona glomerulosa (ZG) from the zona fasciculata/reticularis (ZF/R). The adrenal medulla (M) was also collected for the study [14,50].

#### 4.2.2. Effects of Gonadectomy and Testosteone or Estradiol Replacement on the Expression of the *Giot1* Gene in the Adrenal Gland of the Rat

Adult male and female Wistar rats (12 weeks old; body weight: 120–150 g) were used in this experiment. All procedures described herein were approved by the Local Ethics Committee for Animal Research (Poznan, Poland), permission number: LKE—11/2015. All possible efforts were done to minimise the number of animals and their suffering. Anaesthetized rats (as above) were subjected to gonadectomy or sham surgery. Orchiectomy (ORX) was performed via scrotal access while ovariectomy (OVX) by two dorso-lateral incisions. Fourteen days after surgery half of ORX rats was replaced with testosterone (s.c. injection of Testoviron-Depot, Schering AG, Berlin, Germany, 5 mg/100 g body weight) while other half of OVX animals with estradiol (s.c. injection of Estradiol-Depot, Jenapharm, Jena, Germany, 0.5 mg/100 g body weight). Control rats were s.c. injected with 0.2 mL of sesame oil. Doses of administered depot hormones were based on previous reports [25,51,52,53,54]. It is believed that from both compounds either testosterone or estradiol are liberated slowly, providing a physiological hormone levels in gonadectomized rats. As emphasised by Schulte-Beerbühl and Nieschlag [54], increasing the dose of injected testosterone esters appears not to affect the maximal concentrations of testosterone in the blood but rather the duration of the effect. Moreover, administration of depot compounds allows avoiding the stress evoked by daily administration of the tested substances. After 2 weeks (4 weeks post-surgery), rats were decapitated. Adrenal glands were collected in RNAlater and stored at −70 °C for further analyses. Seminal vesicles and uteri were also collected and weighed.

### 4.3. Primary Adrenocortical Cell Culture

The method of culturing rat adrenocortical cells was described earlier [50,55]. Briefly, adrenals were obtained from forty 20 to 22 day-old male Wistar rats. The glands were immediately transferred into vessel with culture medium (DMEM/F12—Dulbecco’s modified Eagle’s medium without phenol red, Sigma-Aldrich, St. Louis, MO, USA) and mechanically chopped. Tissue fragments were dissociated to cell suspensions using enzymatic digestion in DMEM/F12 supplemented with collagenase type I (1 mg/mL, in water bath at 37 °C for 30 min). The suspension was further mechanically disintegrated using glass pipette and then poured through a nylon filter into a test tube and centrifuged for 10 min at 200× *g*. The collected cells were then suspended in DMEM containing 10% fetal bovine serum (FBS, F7524, Sigma-Aldrich, St. Louis, MO, USA) and 1 mL/100 mL of Antibiotic-Antimycotic Solution (A5955, Sigma-Aldrich, St. Louis, MO, USA). Cells were counted, and their suspensions were placed in 24-well dishes (NUNC Brand Products, Roskilde, Denmark) (1 × 10^4^/well). The culture was incubated in 37 °C and 5% CO^2^. Culture medium was changed every 24 h. At day 3 of culture medium was enriched with test substances including hCG (10^−7^ M), ACTH (10^−7^ M) or H-89 (10^−5^ M). After 2 and 24 h incubation, the medium was collected and stored frozen at −36 °C until hormones were assayed. Cell film from the bottom of wells was processed for subsequent RNA isolation with qPCR and microarray analyses.

### 4.4. RNA Isolation

From collected cells, adrenal compartments (ZG, ZF/R and M) and samples of entire adrenal glands, total RNA was extracted using TRI Reagent (Sigma, St. Louis, MO, USA) and then purified on columns (Rnasy Mini Kit, Qiagen, Hilden, Germany). The total mRNA was determined by optical density at 260 nm, and its purity was estimated by 260/280 nm absorption ratio (higher than 1.8) (NanoDrop spectrophotometer, Thermo Scientific, Waltham, MA, USA). From each RNA sample, 100 ng of total RNA was used for microarray experiments, and remaining RNA was used for qPCR study.

### 4.5. Reverse Transcription

Reverse transcription was performed using Transcriptor High Fidelity cDNA Synthesis Kit with Oligo dT (Roche Holding AG, Basel, Switzerland) in thermal cycler (Biometra UNO II, Gottingen, Germany). All the primers were designed by Primer 3 software (Whitehead Institute for Biomedical Research, Cambridge, MA, USA) (Table 1). The primers were purchased from the Laboratory of DNA Sequencing and Oligonucleotide Synthesis, Institute of Biochemistry and Biophysics, Polish Academy of Sciences, Warsaw, Poland.

### 4.6. Microarray RNA Analysis

Microarray expression analysis was described earlier [14,56,57,58]. RNA subjected to microarray studies was obtained from the above-described experiments. Total RNA (100 ng) from each sample was subjected to two rounds of sense cDNA amplification (Ambion^®^ WT Expression Kit, Ambion, Austin, TX, USA). Obtained cDNA was used for biotin labelling and fragmentation by Affymetrix GeneChip^®^ WT Terminal Labeling and Hybridization kit (Affymetrix, Santa Clara, CA, USA). Biotin-labelled fragments of cDNA (5.5 µg) were hybridised to Affymetrix^®^ Rat Gene 2.1 ST Array Strip (45 °C/20 h). Each array comprised of more than 720,000 unique 25-mer oligonucleotide probes, which included over 27,000 genes. Up to 22 unique probes sequences were hybridised to a single transcript. After hybridisation, every array strip was washed and stained by Fluidics Station of GeneAtlas System (Affymetrix). The array strips were scanned by Imaging Station from GeneAtlas System. Preliminary analysis of the scanned chips was performed using Affymetrix GeneAtlas TM Operating Software (Affymetrix, Santa Clara, CA, USA). The quality of gene expression data was checked according to quality control criteria provided by the software. The obtained CEL files were imported into downstream data analysis software. If not otherwise stated, all of presented analysis and graphs were performed by Bioconductor and R programming language [59]. Each CEL file was merged with a description file. To conduct background correction, normalisation and summarization of results, we used Robust Multiarray Averaging (RMA) algorithm [60]. Presented graphs were prepared by ggplot2 package (plotting system for R language).

### 4.7. qPCR

qPCR was performed using a Lightcycler 2.0 instrument version 4.05 software (Roche, Basel, Switzerland). Using the primers mentioned above, SYBR green detection system was applied as described earlier [56,57,61,62,63,64]. Every of 20 μL reaction mixtures contained 4 μL template cDNA, 0.5 µM of specific primer and a previously determined optimum MgCl_2_ concentration (3.5 M for one reaction). LightCycler Fast Start DNA Master SYBR Green I mix (Roche, Basel, Switzerland) was used. The real-time PCR program included 10 min denaturation step to activate the Taq DNA Polymerase, followed by a three-step amplification program: denaturation at 95 °C for 10 s, annealing at 56 °C for 5 s, and extension at 72 °C for 10 s. Specificity of reaction products was checked by determination of melting points (0.1 °C/s transition rate). Expression of studied genes was related to *B2m*.

### 4.8. Promoter Analysis

One thousand nucleotides lengths of *Giot1* promoter sequence was obtained using Genomic Features and BSgenome.Rnorvegicus.UCSC.rn5 Bioconductor libraries (available online: https://bioconductor.org/packages/release/data/annotation/html/BSgenome.Rnorvegicus.UCSC.rn5.html). Frequency matrix of CREB1 (CAMP responsive element binding protein 1) was downloaded from Jaspar—a database of transcription factor binding profiles (id: MA0018.2, http://jaspar.genereg.net/matrix/MA0018.2/ on 14 May 2018). Subsequently, using the MotifDb Bioconductor library, the potential CREB1 binding sequences were determined. Perfect percentage matching limit was set at 80% similarity to the CREB1 frequency matrix. A similar approach was used for the human *ZNF461* gene, which is considered to be a homologue of rat *Giot1* gene. All *ZNF461* splice variants were retrieved from the TxDb.Hsapiens.UCSC.hg38.knownGene library (available online: https://bioconductor.org/packages/release/data/annotation/html/TxDb.Hsapiens.UCSC.hg38.knownGene.html). From these variants 1000 nt. promoter sequences were obtained, which were analyzed for potential CREB1 binding sequences. The structure of individual *ZNF461* splice variants was presented using GenomicFeatures and Gviz packages (available online: https://bioconductor.org/packages/release/bioc/html/GenomicFeatures.html, https://bioconductor.org/packages/release/bioc/html/Gviz.html).

### 4.9. Multiple Sequence Alignment

Protein sequences GIOT1, ZNF461 from various species were taken from UniProt database (https://www.uniprot.org). GIOT2 rat protein and human ZNF460 were also extracted. Afterwards protein sequences were uploaded to Clustal Omega aligner program (https://www.ebi.ac.uk/Tools/msa/clustalo/). Multiple sequence alignment was performed in two versions. In the first one, all of the obtained sequences were compared each other, in the second analysis, a comparison of the human ZNF461 with the rat GIOT1 was performed. Similar or identical amino acids were determined and mark on the output figure. Additionally, conservative zinc fingers domains were marked.

### 4.10. Analysis of RNA-seq Dataset Obtained from Adrenals of Male and Female rats During Postnatal Ontogenesis

Data on the expression of *Giot1* in adrenal glands of male and female rats during postnatal ontogenesis were obtained from open database. These studies were performed on Fischer 344 strain rats aged 2, 6, 21 and 104 weeks [13]. Experimental data were obtained from Gene Expression Omnibus database, accession number: GSE53960 (available at https://www.ncbi.nlm.nih.gov/geo/query/acc.cgi?acc=GSE53960) (accessed date: 03 August 2018).

### 4.11. Statistics

Multiple group comparisons were performed using one-way analysis of variance (ANOVA) followed by Tukey’s HSD test. Calculation was made using R environment with multcomp library. Results were considered as statistically significant when p values from ANOVA were lower than 0.05. In such cases, post-hoc Tukey’s HSD test was performed. Statistical significance level of post-hoc test was set to 5%. Results of Tukey’s HSD test presented in figures were marked by letters. Groups sharing the same letter are not significantly different according to Tukey’s HSD test. In the case of two group comparisons, a statistical evaluation of the differences was carried out using the Student’s *t*-test with asterisk annotation (* *p* < 0.05; ** *p* < 0.02; *** *p* < 0.01). Association between expression of *Giot1* and *Nr4a1* was tested using Pearson correlation test. Normalized log2 signal intensity values from microarray were presented as scatter plots with a line of best fit. Confidence interval level for predictions based on linear models was set at 95%. Each of the described experiments were repeated at least three times.

## 5. Conclusions

Our study found that ACTH rapidly stimulates *Giot1* expression, but hCG does not affect the *Giot1* gene expression, presumably due to the low expression of gonadotropin receptor in adrenals. Both testosterone and estradiol administered in vivo have an inhibitory effect on the expression of the *Giot1* gene in the rat adrenals. We also demonstrate that apart from the triggering factor, such as ACTH in the case of adrenal glands, the intracellular mechanism of *Giot1* gene regulation in adrenals is identical to that in the gonads. The cAMP-dependent signaling pathway regulates expression of *Giot1* through activation of the NR4A1 transcription factor. However, further studies are required to explain the *Giot1* role in adrenal gland using the knockdown and/or overexpression models of *Giot1* gene in rat adrenals.

## Figures and Tables

**Table 1:** Primers used for qPCR validation of *Giot1* gene. *B2m* (beta-2 microglobulin) was the reference gene.

Gene Symbol	Genbank Accession Number	Primer	Primer Sequence (5′-3′)	Position	PCR Product Size (bp)
*B2m*	NM_012512.2	S A	CTTGCAGAGTTAAACACGTCA CTTGATTACATGTCTCGGTC	316-336 366-385	70
*Giot1*	NM_133563.1	S A	AATAGGAGGGGACACTTCCG CATCCTCATAGGTGACTGCAT	152-171 294-314	163

## Supplementary Materials

Supplementary materials can be found at http://www.mdpi.com/1422-0067/19/8/2285/s1.

## Abbreviations

ACTH	Adrenocorticotropic hormone
HCG	Human chorionic gonadotropin
GIOT1	Gonadotropin-inducible ovarian transcription factor-1
cAMP	Cyclic adenosine monophosphate
Star	Steroidogenic acute regulatory protein
Cyp11a1	Cytochrome P450 family 11 subfamily A member 1
HSD3B2	3β-hydroxysteroid dehydrogenase type 2
SF-1	Steroidogenic factor-1
DAX-1	Dosage-sensitive sex reversal, adrenal hypoplasia critical region, on chromosome X, gene 1
PMSG	Pregnant mare’s serum gonadotropin
FSH	Follicle-stimulating hormone
PKA	Protein kinase A
CRE	cAMP response element
LHCGR	Luteinizing hormone/choriogonadotropin receptor
MC2R	Melanocortin 2 receptor
RPKM	Reads Per KilobaseMilion
LH	Luteinizing hormone
ORX	Orchiectomy
OVX	Ovariectomy
CREB1	cAMP responsive element binding protein 1
KRAB-A	Krüppel associated box- A
ZF/R	Zona fasciculata/reticularis
ZG	Zona glomerulosa
M	Medulla
ATP	Adenosine triphosphate
HPA	Hypothalamic-pituitary-adrenal
CRH	Corticotropin-releasing hormone
DHEA	Dehydroepiandrosterone
NGFIB	Nerve growth factor IB
CYP11B2	Cytochrome P450 family 11 subfamily B member 2
FBS	Fetal bovine serum
*B2m*	Beta-2 microglobulin
RMAZNF461	Robust Multiarray AveragingZinc finger protein 461
