# Supplementary material for: Effect of ACTH and hCG on the Expression of Gonadotropin-Inducible Ovarian Transcription Factor 1 (Giot1) Gene in the Rat Adrenal Gland
Source: Int J Mol Sci. 2018 Aug 3;19(8):2285. doi: 10.3390/ijms19082285 (PMC6121328; doi:10.3390/ijms19082285)
Supplement: Supplementary file 1 [file ijms-19-02285-s001.pdf]

International Journal of
Molecular Sciences

MDPI

1. SUPLEMENT

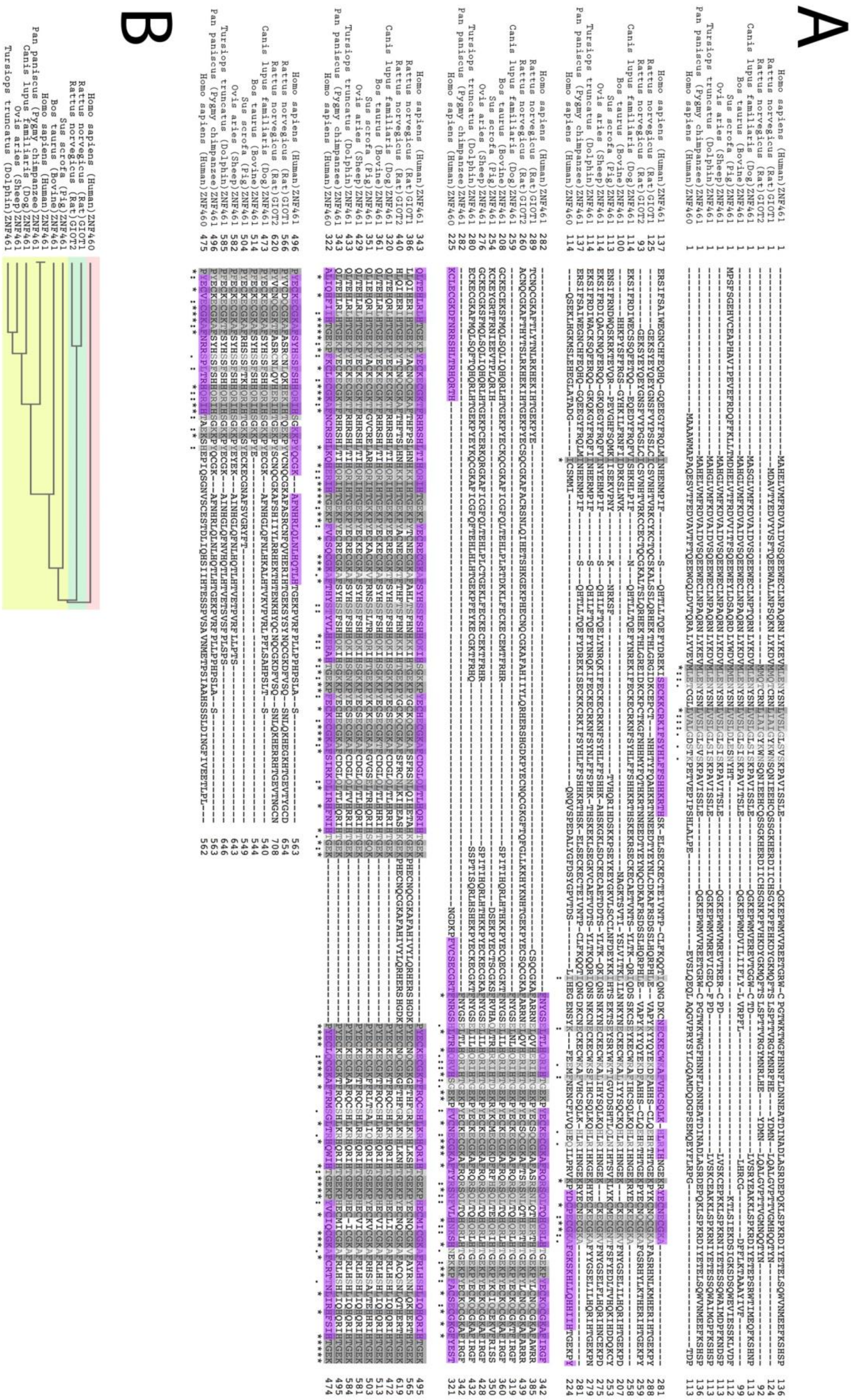

Figure S1. (A) Multiple sequence alignment of the following protein sequences: Homo sapiens (Human) ZNF461, Rattus norvegicus (Rat) GIOT1, Rattus norvegicus (Rat) GIOT2, Canis lupus familiaris (Dog) ZNF461, Bos taurus (Bovine) ZNF461, Sus scrofa (Pig)ZNF461, Ovis aries (Sheep) ZNF461, Tursiops truncatus (Dolphin) ZNF461, Pan paniscus (Pygmy chimpanzee) ZNF461 and Homo sapiens (Human) ZNF460. Identical sequences are marked with a dark grey colour and an asterisk. Similar sequences are indicated by a light grey colour and a colon or dot depending on the degree of similarity. Conservative zinc fingers domains were marked in purple. (B) phylogenetic tree generated from a multiple sequence alignment. Three independent clusters are presented in different colours.
